# UK Chickpea Consumption Doubled from 2008/09–2018/19

**DOI:** 10.3390/nu15224784

**Published:** 2023-11-15

**Authors:** Inga Kutepova, Colin D. Rehm, Samara Joy Friend

**Affiliations:** 1 Life Sciences, PepsiCo R&D, Reading RG2 6UW, UK; 2 Life Sciences, PepsiCo R&D, Purchase, NY 10577, USAsamarajoy.friend@pepsico.com (S.J.F.)

**Keywords:** chickpea, National Diet and Nutrition Survey, United Kingdom

## Abstract

**Background:** Only 9% of individuals in the United Kingdom (UK) meet the recommendation for dietary fibre intake. Little is known about chickpea consumption in the UK. **Methods:** Chickpea intake trends and sociodemographic patterns were analysed using the National Diet and Nutrition Survey Rolling Programme data collected from 2008/09 to 2018/19 among 15,655 individuals ≥1.5 years completing a four-day food diary. Chickpea consumers were identified based on a list of chickpea-containing foods, with the most consumed foods being hummus, boiled chickpeas, chickpea flour, and low/reduced-fat hummus. Micronutrient and food group intakes were compared between chickpea consumers and non-consumers; the Modified Healthy Dietary Score was also assessed, which measures adherence to UK dietary recommendations. **Results:** Chickpea consumption increased from 6.1% (2008–2012) to 12.3% (2016–2019). Among 1.5–3 years, consumption increased from 5.7% to 13.4%, and among 19–64 years, consumption increased from 7.1% to 14.4%. The percentage of individuals eating chickpeas was higher among individuals with higher incomes and more education. Healthy-weight adults were more likely to consume chickpeas compared to those who were overweight or obese. Compared to both bean and non-bean consumers, chickpea consumers ate significantly more dietary fibre, fruits and vegetables, pulses, nuts, and less red meat and processed meat products. Chickpea consumers also had a higher Modified Healthy Dietary Score. **Conclusions:** In the UK, chickpea consumption more than doubled from 2008/09 to 2018/19. Chickpea consumers had a higher diet quality than non-consumers.

## 1. Introduction

Pulses, which are part of the legume family, are edible seeds grown in pods and include lentils, chickpeas, beans, and peas [1]. The United Kingdom (UK) Eatwell Guide recommends consuming more protein from beans and pulses, since they are low in fat and dense in protein, fibre, vitamins, and minerals, making them a good alternative to meat [1,2,3]. To promote a healthier dietary pattern, it is recommended to decrease the consumption of saturated fat and increase intake of dietary fibre, such as by substituting a portion of red meat in one’s diet with pulses, allowing individuals to choose from chickpeas, black-eyed peas, mung beans, and other pulses [1]. One serving of pulses, such as chickpeas, is defined as three heaping tablespoons (~80 g) [4,5]. This contributes about 5.7 g of fibre and 6.7 g of protein [6], while also counting towards one of five servings of fruits and vegetables per day [4,6,7].

The UK Scientific Advisory Committee on Nutrition (SACN) observed a substantial reduction in the risk of cardiovascular disease, type 2 diabetes, and colorectal cancer with a daily fibre intake ≥30 g [8]. Despite this recommended value, the average daily intake of fibre in adults (19–64 years) in the United Kingdom is 19.7 g, and only 9% meet the recommendations for fibre [9]. An increase in legume intake may contribute to higher fibre consumption [2,10,11,12,13]. Dietary patterns that prioritise the consumption of plant-based foods, particularly beans and legumes, promote overall well-being and contribute to the prevention and management of several nutrition-related chronic diseases [2,11,12,14].

Chickpeas are a type of pulse that belongs to the legume family and have been traditionally used in a variety of dishes, including curries, because of their flavour and broad sensory applications. In addition to being a source of high-quality protein and dietary fibre, chickpeas also provide micronutrients, particularly folate, vitamin B6, iron, and magnesium [5]. Hummus, which is generally consumed as a dip but also as a condiment, contributes to chickpea consumption [10,11,15].

To our knowledge, no information is available on UK chickpea consumption patterns. Therefore, we examined nationally representative population-based survey data collected to examine trends in chickpea consumption, as well as describing the health, nutrition, and dietary patterns of UK chickpea consumers.

## 2. Materials and Methods

### 2.1. Data Source and Population

National Diet and Nutrition Survey (NDNS) data from 2008–2019, encompassing years 1–11 of the rolling programme, were analysed for this study. The data included 15,655 individuals aged ≥1.5 years. NDNS is a continuous cross-sectional nationally representative survey covering all four countries: England, Scotland, Wales, and Northern Ireland [16].

### 2.2. Dietary Assessment Methodology

For each individual, food diary data was collected for four consecutive days, including one weekend day. Computer-assisted randomly allocated four days, including one weekend day, to ensure every day of the week was equally represented. Each food diary provided sample sheets suitable for the respective age group, illustrating the proper way to fill out the diary and the level of detail required. The interviewers conducted three visits with each participant: to provide food diary; to assess compliance and respond to questions (a phone call could replace a home visit), and to gather and review the diary. Interviewers edited possible omissions and verified the diary records, which were then coded by trained coders.

### 2.3. Classification of Chickpea and Bean Consumers

A list of all chickpea-containing foods in the NDNS nutrient database was identified by the study authors. Individuals were classified as a chickpea consumer or not based on whether they consumed any of these foods in the four days of data collection. The most consumed chickpea-containing items were hummus, boiled chickpeas, chickpea flour, and low/reduced-fat hummus. Individuals were also classified as hummus consumers or other chickpea consumers; an individual could be classified as both a hummus and other chickpea consumer. Bean consumers were those that consumed beans but did not consume chickpeas. A non-bean consumer was an individual that did not consume any beans or chickpeas.

### 2.4. Dietary Variables

This study compared nutrient and food group intakes between chickpea consumers, bean consumers, and non-bean consumers. The macronutrients (all in g/day except for kilocalories) evaluated were calories, protein, total carbohydrate, free sugars, dietary fibre, total fat, saturated fat, monounsaturated fatty acid, and polyunsaturated fatty acid (PUFA). The micronutrients evaluated were potassium (mg/d), vitamin D (µg/d), iron (mg/d), magnesium (mg/d), sodium (mg/d), and calcium (mg/d). The food groups analysed were fruits and vegetables, pulses and nuts, red and processed meat, fish, and oily fish (all in g/d).

The Modified Healthy Dietary Score (mHDS) consists of 14 components: saturated fat, PUFA, protein, carbohydrates, dietary fibre, fruits and vegetables, pulses and nuts, free sugars, *trans* fat, fish, oily fish, red meat and processed meat, calcium, and sodium. Macronutrient scores for the mHDS were energy-adjusted, and each component receives a score of one or zero based on meeting the pre-specified cutoff value. The possible mHDS range from 0 to 14, with higher values indicating increased adherence to dietary recommendations [17].

### 2.5. Covariates

The covariates analysed were sex, age, ethnic group, income, education, body mass index (BMI), vegetarian-type dietary pattern, cigarette smoking status, and self-reported health status. The age groups were 1.5–3 years, 4–10 years, 11–18 years, 19–64 years, and ≥65 years. Ethnic group was categorised as white and other due to small sample sizes for the Mixed ethnic group, Black or Black British, Asian or Asian British. Equivalised household income terciles were calculated by creating a score for each household (based on the number, age, and relationships of adults and children in the household) and then dividing the total household income by this score. Education was defined as the age when education was completed: low ≤16 years, moderate 17–18 years, and high ≥19 years. Education data were only analysed for adults. Adult BMI was categorised as healthy (18.5–24.9 kg/m^2^), overweight (25–29.9 kg/m^2^), and obese (≥30 kg/m^2^). Individuals consuming a vegetarian dietary pattern were identified based on reporting consumption of no red meat or poultry in their food diary. Cigarette smoking status was grouped as never, former, or current. Self-reported health status was categorised as very good, good, fair, and bad/very bad.

The proportion of individuals consuming chickpeas was analysed by sociodemographic characteristics, including age, sex, ethnic group, education, and income. Trends in the percent of individuals consuming chickpeas between 2008 and 2019 were analysed overall and stratified by the same sociodemographic characteristics. The percent of individuals consuming chickpeas by selected health characteristics, including BMI, vegetarian-type dietary pattern, self-reported health status, and cigarette smoking status, was also analysed. A comparison of dietary variables and modified Healthy Dietary Score (mHDS) among chickpea consumers, bean consumers, and non-bean consumers, as well as a comparison of mHDS components, was also conducted. For all non-trend analyses, data from 2016/2019 were used to represent the most recently available snapshot of patterns.

All analyses were performed in SAS 9.4 using appropriate survey sampling weights, strata, and survey commands. Tests for linear trend were determined from fitting a survey-weighted linear regression model with ordered categorical variables as independent variables. Tests for interactions in the linear trend were assessed via a multiplicative interaction term between survey cycle and the categorical variable of interest (e.g., age group, gender, or income). An alpha-level of 0.05 was used to determine statistical significance, and as a descriptive analysis, there were no adjustments for multiple comparisons.

## 3. Results

In the United Kingdom, the percentage of individuals consuming chickpeas increased from 6.1% (95% confidence interval (CI): 5.2, 7.0) in 2008–2012 to 12.3% (10.8, 13.8) in 2016–2019 (Table 1). Consumption of chickpeas increased across all age groups but was not statistically significant for those 4–10 years (*p*-trend = 0.06). The largest absolute increase was amongst those 1.5–3 years, from 5.7% (3.7, 7.7) in 2008–2012 to 13.4% (9.9, 16.8) in 2016–2019. Among those 19–64 years, 7.1% (5.8, 8.5) consumed chickpeas in 2008–2012, increasing to 14.4% (12.1, 16.6) in 2016–2019. Trends for hummus and other chickpea separately are shown in Supplementary Table S1. Briefly, there was an increase in consumption of both hummus and other chickpeas from 2008/12 to 2016/19, increasing from 3.4% to 6.0% and 3.3% to 8.7%, respectively. The increase in chickpea consumption among young children was almost exclusively driven by increasing hummus consumption.

Analysis by ethnic group indicates that a higher proportion of non-white individuals consumed chickpeas (18.5% (13.6, 23.4)) than white individuals 11.3% (9.8, 12.9). A greater percentage of high-income individuals (16.9% (13.8, 20.0)) consumed chickpeas than lower-income individuals (9.9% (7.2, 12.5)). Similarly, among adults with more education, 21.6% (17.8, 25.3) consumed chickpeas while 6.6% (4.5, 8.7) consumed chickpeas among individuals with lower levels of education. While overall chickpea consumption followed an income gradient, this appears to be driven by hummus alone. For education, there appeared to be a gradient for both hummus and other chickpea. Supplementary Table S2 shows a breakdown of chickpea consumption compared to bean consumption and non-bean consumption.

Among adults, chickpea consumption was also analysed by BMI, vegetarian dietary pattern, cigarette smoking status, and self-reported health status in 2016–2019 (Table 2). Chickpea consumption was highest among adults with a healthy weight 15.7% (12.2, 19.2) than among overweight or obese adults, 13.0% (10.0, 16.0) and 11.1% (7.8, 14.5), respectively. Among adults consuming a vegetarian-type dietary pattern, 37.8% (30.4, 45.2) consumed chickpeas compared to 10.9% (9.1, 12.7) for those consuming a non-vegetarian-type pattern. Sixteen percent (13.7, 19.0) of never smokers consumed chickpeas compared to only 6.5% (4.3, 8.8) of current smokers. Twice as many (16.7% (13.4, 20.0)) adults who reported very good health status consumed chickpeas compared to 8.4% (7.7, 9.1) of adults who reported bad/very bad health status.

The diets of chickpea consumers, bean consumers, and non-bean consumers differed by specific nutrients and food groups, with chickpea consumers generally consuming more nutrients and food groups to encourage, and fewer dietary constituents to limit. All differences described below are significant at the 0.05 level when comparing chickpea consumers to the other two groups. Chickpea consumers consumed more dietary fibre (23.1 g (chickpea consumers) vs. 19.4 g (bean consumers) vs. 15.8 g (neither)) than both bean consumers and non-bean consumers (Table 3). Chickpea consumers also consumed more MUFA (26.6 g vs. 24.4 g vs. 23.4 g) and PUFA (11.5 g vs. 9.5 g vs. 8.7 g) than bean consumers or non-bean consumers. Chickpea consumers also consumed more fruits and vegetables (361.6 g vs. 271.9 g vs. 235.6 g), more pulses and nuts (42.8 g vs. 28.7 g vs. 3.5 g), and less red meat and processed meat products (24.4 g vs. 45.4 g vs. 43.1 g) than both bean and non-bean consumers. Supplementary Table S3 shows the multivariable-adjusted means demonstrating that the patterns observed above were not explained by adjusting for age, sex, ethnicity, income, and education. The association between chickpea consumption and dietary intakes, among adults only, was comparable to the total population (Supplementary Tables S4 and S5). Children could not be analysed separately due to limited sample size.

**Figure 1:**
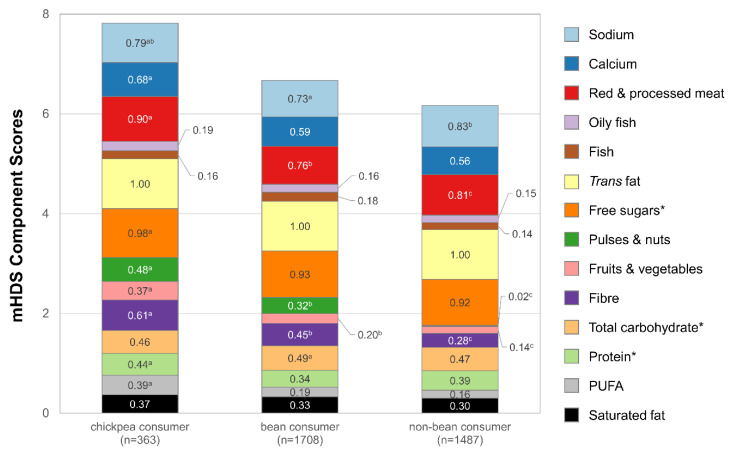
Mean Modified Healthy Dietary Score (mHDS) among chickpea consumers, bean consumers who did not consume chickpeas, and individuals consuming neither. Figure footnotes: Asterisks indicate that the component of mHDS is energy-adjusted. Differing letters indicate that values are different at the *p* < 0.05 level across each category. If there is no letter, then the value is not significantly different from any of the other values.

As expected, based on the patterns above, chickpea consumers had a higher mHDS (7.8 vs. 6.7 vs. 6.2) than both bean consumers and non-bean consumers. For specific mHDS components, the estimate can be interpreted as the proportion of individuals in each category meeting recommended intake levels for chickpea consumers, bean consumers, and non-bean consumers (Figure 1). The proportion of chickpea consumers consuming less than the recommended amounts of red and processed meat was statistically significantly higher for chickpea consumers versus both bean and non-bean consumers (90% vs. 76% vs. 81%). In addition, fruit and vegetable consumption (61% vs. 45% vs. 28%) and dietary fibre consumption (37% vs. 20% vs. 14%) were significantly higher for chickpea consumers.

## 4. Discussion

The proportion of UK individuals consuming chickpeas more than doubled from 2008/09 to 2018/19, from 6.1% and 12.3%. The percentage of individuals consuming chickpeas increased the most among children aged 1.5–3 years, primarily due to hummus. Additionally, it was observed that there was an increase in the percentage of individuals aged 65+ consuming chickpeas. This trend is particularly noteworthy considering that research indicates a positive correlation between a higher intake of legumes and improved longevity in the elderly, irrespective of their ethnic background [18]. Chickpea consumption was more common among non-white individuals than white individuals. This may be because chickpeas are commonly used in the cuisine of individuals from South Asia and the Caribbean, groups that make up a large proportion of the non-white population in the UK [19]. Despite this, the percentage consuming chickpeas increased in the white population, whereas non-whites’ intake remained relatively unchanged. Therefore, the change in chickpea consumption at the total population level was mainly due to a change in the consumption pattern of white individuals. The consumption of hummus is more prevalent among those with a higher than lower income, whereas this distinction does not apply to other forms of chickpea. The reasons for these differences are uncertain but may be due to the cost of other chickpea products compared to hummus, and future consumer-oriented research could further explore this observation. Consumption of chickpeas was more prevalent among those with more education, better health status, and healthy weight. Chickpea consumption may be one of the indicators of better health and diet quality; however, there is a possibility of self-selection whereby individuals focusing on their health may be more inclined to consume chickpeas since they were also less likely to report smoking.

Despite trends in the UK diet showing modest improvement (e.g., lower sodium, free sugars, and increased nuts), aside from the two-fold increase in chickpea consumption, we do not see many trends as strong as those observed in the present study [20,21]. Comparable data from other countries on chickpea consumption patterns and trends are limited. A US-based study observed a similar two-fold increase in the proportion of chickpea consumers from 1.9% in 2003–2006 to 4.5% in 2015–2018, though the proportion consuming chickpea cannot be directly compared as this study used two 24 hour dietary recalls, while our study used a four-day food diary [10].

Between 2008 and 2019, there was an increase in global chickpea production from 8.6 M to 12.4 M tonnes of processed crops, far outpacing population growth [22]. The Mintel Global New Products Database shows a considerable increase in the number of new product launches that contain chickpea in Europe. For example, in the UK, there were 96 new chickpea-containing items launched in 2008 compared to 252 in 2019 [23]. A comparable pattern was observed in nearly all large European countries, although the UK market had the largest increase. Increased consumption of products with chickpea may be due to consumer demand, but it is also possible that increased market variety prompted consumers to try new products and diversify their diet.

Whilst chickpea consumption has historically been associated with cultural dietary patterns from the Mediterranean, Middle East, and South Asia, contemporary consumption patterns, such as increasing interest in plant-based dietary patterns, may impact chickpea consumption. Chickpeas can be a valuable source of plant-based protein, and chickpea’s protein quality is better than other beans, which is important for individuals relying on plant sources of proteins [19]. Between 2008–2019, the percentage of the population consuming a vegetarian-type dietary pattern increased from 4.8% (2008–2012) to 7.4% (2017–2019) among adults. The proportion consuming chickpeas was higher among individuals with a vegetarian dietary pattern than the proportion among non-vegetarians. Vegetarian-type dietary patterns have almost doubled, which may explain part or some of the increase in chickpea consumption.

According to the Global Burden of Disease study, among 15 dietary risks evaluated in the UK, low intake of legumes is associated with the 5th most Disability Adjusted Life Years, trailing only low whole grains, high processed meat, low fruit, and high red meat dietary risks. Low intake of legumes is associated with more ill health than sub-optimal consumption of nuts/seeds, sodium, vegetables, and sugar-sweetened beverages (individually, not collectively). Low fibre consumption is also the 7th leading dietary risk in the United Kingdom [24]. This means that increasing legume consumption and fibre intake in the United Kingdom might have major population health benefits [25,26,27]. In our analysis, chickpea consumers consumed more legumes, as one would expect, but also consumed more fruits and vegetables, nuts, more unsaturated fat, more calcium, and less red and processed meat. All these dietary factors are associated with positive health outcomes, and the chickpea consumers had favourable intakes for each [28]. Not all these observed associations are the direct result of consuming chickpeas but are driven by a combination of consuming a dietary pattern that contains chickpea (e.g., chickpea-containing foods often contain vegetables or nuts/seeds, for example). Alternatively, individuals who chooses to consume chickpea are also likely to consume more dietary constituents that promote health (e.g., chickpea consumers are more likely to consume fruits because they are of higher socioeconomic status or more generally concerned with issues related to health). These results are generally comparable to a recently published study in the US, which also found that chickpea consumers were more likely to consume whole grains, nuts/seeds, whole fruit, and less red meat and processed meat [10]. Interestingly, in the US study, chickpea consumers consumed less added sugar than non-consumers, while no association was observed in the UK for free sugars. Additionally, in the US, chickpea consumption was not associated with vegetable consumption. While chickpea consumers did have more favourable diets compared to non-legume consumers and consumers of other types of legumes, their diets were still sub-optimal in many regards, particularly in terms of fibre intake, where we observed an average intake of 21 g/d, which remains far below the 30 g/d value widely recommended [7]. This suggests that aligning the UK diet with recommendations will require further changes beyond simply adopting the dietary patterns of chickpea consumers or adding chickpea to the diet.

The strengths of this study are that UK NDNS uses a four-day food diary, including a weekend day, which allows us to reasonably capture day-to-day variation in the diet and identify the consumption of chickpea-containing foods. Further, NDNS is designed to be nationally representative and includes individuals of all ages and includes detailed sociodemographic and other health data. Limitations of a four-day food diary include the possibility that some individuals alter or simplify their diet, leading to both random and systematic errors in the dietary intake data [29]. Relatedly, it is possible individuals may forget to report certain foods or may purposely omit certain foods, and that such misreporting is systematically impacted by participant characteristics. Lastly, while NDNS is generally representative of the overall population, the sample size of non-white individuals was relatively small, hindering analyses of specific non-white population groups (e.g., British Asian).

## 5. Conclusions

We found that the proportion of the population consuming chickpeas doubled in the United Kingdom and that consumption was associated with generally better diet quality. Chickpea is a source of high-quality protein and dietary fibre and might provide potential health benefits. Increasing chickpea consumption may help increase fibre intake and improve diet quality. Future research may examine different ways to increase fibre consumption, including chickpea consumption.

## Figures and Tables

**Table 1:** Trends in UK chickpea consumption overall and according to sociodemographic characteristics, 2008–2019.

	Chickpea Consumers, % (95% Confidence Interval)					
	2008–2012 (*n* = 6828)	2012–2014 (*n* = 2546)	2014–2016 (*n* = 2723)	2016–2019 (*n* = 3558)	*p*-Trend	*p*-Interaction
						
Total	6.1 (5.2, 7.0)	8.2 (6.7, 9.8)	11.7 (9.9, 13.5)	12.3 (10.8, 13.8)	<0.001	-
Age group, year						
1.5–3	5.7 (3.7, 7.7)	4.8 (1.6, 8.0) ^1^	12.5 (8.5, 16.4)	13.4 (9.9, 16.8)	<0.001	0.50
4–10	7.1 (5.4, 8.9)	7.2 (4.4, 9.9)	9.9 (6.8, 13.1)	8.8 (6.3, 11.3)	0.06	
11–18	4.4 (3.0, 5.9)	2.8 (1.0, 4.5) ^1^	5.5 (3.2, 7.7)	10.3 (7.4, 13.2)	<0.001	
19–64	7.1 (5.8, 8.5)	10.1 (7.8, 12.4)	14.7 (12.0, 17.4)	14.4 (12.1, 16.6)	<0.001	
≥65	3.0 (1.5, 4.6)	5.8 (2.7, 8.9)	5.6 (2.5, 8.7)	8.1 (5.2, 10.9)	<0.001	
Sex						
Male	5.0 (3.8, 6.2)	6.6 (4.4, 8.9)	9.4 (7.0, 11.8)	10.9 (8.8, 12.9)	<0.001	0.60
Female	7.2 (5.9, 8.6)	9.8 (7.6, 11.9)	14.0 (11.2, 16.8)	13.7 (11.6, 15.8)	<0.001	
Race/ethnicity						
White	4.8 (4.0, 5.7)	6.7 (5.1, 8.2)	10.3 (8.4, 12.1)	11.3 (9.8, 12.9)	<0.001	0.002
Other *	15.9 (11.7, 20.1)	19.9 (13.9, 25.8)	19.4 (13.3, 25.4)	18.5 (13.6, 23.4)	0.26	
Income Tertile **						
Low	3.8 (2.4, 5.2)	7.7 (4.8, 10.6)	7.8 (5.0, 10.6)	9.9 (7.2, 12.5)	<0.001	0.04
Medium	5.5 (3.9, 7.0)	5.9 (3.2, 8.6)	10.0 (6.5, 13.6)	10.8 (8.2, 13.5)	<0.001	
High	9.4 (7.6, 11.3)	10.7 (7.9, 13.6)	15.8 (12.4, 19.1)	16.9 (13.8, 20.0)	<0.001	
Education (adults) ***						
Low (≥16 years)	3.1 (2.0, 4.1)	4.7 (2.5, 6.9)	4.8 (3.0, 6.6)	6.6 (4.5, 8.7)	<0.001	0.44
Medium (17–18 years)	6.0 (3.8, 8.2)	11.3 (6.6, 16.0)	11.2 (7.1, 15.2)	10.3 (7.1, 13.6)	0.02	
High (≥19 years)	11.5 (8.7, 14.3)	14.8 (10.8, 18.9)	23.1 (18.1, 28.1)	21.6 (17.8, 25.3)	<0.001	

^1^ Interpret with caution due to relative standard error exceeding 30%. * Other includes Mixed ethnic group, Black or Black British, Asian or Asian British, Any other group. These groups were combined because there was not sufficient sample size in each group. ** Equivalised household income terciles were calculated by creating a score for each household (based on the number, age, and relationships of adults and children in the household), and then dividing the total household income by this score to get an equivalised household income. *** Education is the age when education is completed.

**Table 2:** Chickpea consumption by body mass index, vegetarian-type dietary pattern, smoking status, and self-reported overall health status 2016–2019 (adults ≥ 19 years).

	*n*	Chickpea Consumers, % (95% CI)
		
Adults	1844	11.6 (10.1, 13.0)
		
Body mass index, kg/m^2^		
Healthy weight: 18.5–24.9	584	15.7 (12.2, 19.2)
Overweight: 25–29.9	639	13.0 (10.0, 16.0)
Obese: ≥30	439	11.1 (7.8, 14.5)
		
Vegetarian-type dietary pattern ^a^		
No	1720	10.9 (9.1, 12.7)
Yes	120	37.8 (30.4, 45.2)
		
Cigarette smoking status		
Current	312	6.5 (4.3, 8.8)
Former	443	8.5 (6.0, 11.0)
Never	1061	16.3 (13.7, 19.0)
		
Self-reported overall health status		
Very good	608	16.7 (13.4, 20.0)
Good	801	12.7 (10.1, 15.4)
Fair	340	7.0 (4.1, 10.0)
Bad/very bad	92	8.4 (7.7, 9.1)
		

^a^ Vegetarian-type dietary pattern is defined as not having consumed meat or poultry on any of the four days that dietary data were collected.

**Table 3:** Comparison of dietary intakes among chickpea consumers, bean consumers *, and non-bean consumers, 2016–2019 (youth and adults).

	Mean (95% CI)		
	Chickpea Consumers (*n* = 363)	Bean Consumers (*n* = 1708)	Non-Bean Consumers (*n* = 1487)
Kcal/d	1795 (1745, 1846) ^a^	1757 (1716, 1798) ^a^	1656 (1620, 1691) ^b^
			
Protein, g/d	71.5 (68.6, 74.4) ^a^	72.4 (70.7, 74.2) ^a^	67.0 (65.3, 68.8) ^b^
Carbohydrate, g/d	215 (208, 221) ^a^	216 (210, 221) ^a^	201 (196, 206) ^b^
Free sugar, g/d	45.4 (42.3, 48.5)	48.8 (46.6, 50.1)	47.6 (45.1, 50.2)
Dietary fibre, g/d	23.1 (22.1, 24.2) ^a^	19.4 (18.7, 20.0) ^b^	15.8 (15.4, 16.2) ^c^
Total fat, g/d	71.3 (68.7, 74.0) ^a^	66.5 (64.7, 68.3) ^b^	63.8 (62.1, 65.5) ^c^
Saturated fat, g/d	24.7 (23.6, 25.9)	24.5 (23.8, 25.3)	24.2 (23.4, 24.9)
MUFA, g/d	26.6 (25.4, 27.8) ^a^	24.4 (23.7, 25.1) ^b^	23.4 (22.7, 24.1) ^c^
PUFA, g/d	11.5 (10.9, 12.1) ^a^	9.5 (9.2, 9.8) ^b^	8.7 (8.4, 8.9) ^c^
			
Potassium, mg/d	2878 (2783, 2973) ^a^	2742 (2679, 2805) ^b^	2507 (2451, 2564) ^c^
Vitamin D, μg/d	6.3 (4.7, 7.9)	5.5 (4.4, 6.6)	5.0 (4.0, 6.0)
Iron, mg/d	13.3 (11.1, 15.5) ^a^	11.8 (11.1, 12.5) ^a^	10.3 (9.5, 11.0) ^b^
Magnesium, mg/d	305.5 (291.2, 319.8) ^a^	265.0 (257.2, 271.9) ^b^	235.4 (228.4, 242.4) ^c^
Sodium, mg/d	1943 (1869, 2018) ^a^	2005 (1952, 2058) ^a^	1796 (1746, 1846) ^b^
Calcium, mg/d	849 (813, 885) ^a^	831 (807, 855) ^a^	787 (764, 810) ^b^
			
Food Groups			
Fruits and Vegetables, g/d	361.6 (339.0, 384.6) ^a^	271.9 (260.1, 283.6) ^b^	235.6 (224.2, 247.0) ^c^
Pulses and nuts, g/d	42.8 (37.3, 48.4) ^a^	28.7 (25.1, 32.3) ^b^	3.5 (2.9, 4.2) ^c^
Red and processed meat, g/d	24.4 (20.4, 28.3) ^a^	45.4 (42.5, 48.3) ^b^	43.1 (40.4, 45.9) ^b^
Fish, g/d	19.3 (15.8, 22.9)	19.4 (17.4, 21.3)	19.2 (17.3, 21.2)
Oily fish, g/d	9.1 (6.5, 11.8)	8.2 (6.9, 9.4)	6.6 (5.5, 7.7)
			
Modified Healthy Dietary Score (range 0–14)	7.8 (7.6, 8.0) ^a^	6.7 (6.6, 6.8) ^b^	6.2 (6.1, 6.3) ^c^
			

* A bean consumer consumes beans but not chickpeas. Differing letters indicate that values are different at the *p* < 0.05 level across each row. If there is no letter, then the value is not significantly different from any of the other values.

## Data Availability

Not applicable.

## Supplementary Materials

The following supporting information can be downloaded at: https://www.mdpi.com/article/10.3390/nu15224784/s1, Table S1: Trends in UK hummus and chickpea consumption overall and according to sociodemographic characteristics, 2008–2019; Table S2: UK chickpea and bean consumption overall and according to socio-demographic characteristics, 2016–2019; Table S3: Comparison of dietary intakes among chickpea consumers, bean consumers *, and non-bean consumers, 2016–2019 (youth and adults); Table S4: Comparison of dietary intakes among chickpea consumers, bean consumers *, and non-bean consumers, 2016–2019 (just adults); Table S5: Comparison of dietary intakes among chickpea consumers, bean consumers *, and non-bean consumers among adults only, 2016–2019.
