# Supplementary material for: UK Chickpea Consumption Doubled from 2008/09–2018/19
Source: Nutrients. 2023 Nov 15;15(22):4784. doi: 10.3390/nu15224784 (PMC10675415; doi:10.3390/nu15224784)
Supplement: Supplementary file 1 [file nutrients-15-04784-s001.zip › nutrients-2701914-supplementary.pdf]

Online Supplementary Materials

Table S1. Trends in UK hummus and chickpea consumption overall and according to sociodemographic characteristics, 2008-2019

	Hummus, % (95% CI)				Other chickpea, % (95% CI)			
	2008-12	2012-14	2014-16	2016-19	2008-12	2012-14	2014-16	2016-19
Total	3.4 (2.7, 4.1)	4.1 (3.0, 5.2)	6.1 (4.8, 7.5)	6.0 (5.0, 7.1)	3.3 (2.6, 3.9)	4.4 (3.2, 5.6)	7.4 (5.8, 8.9)	8.7 (7.4, 10.0)
Age group, y								
1.5-3	4.2 (2.4, 6.0)	3.4 (0.3, 6.5)	7.6 (4.4, 10.8)	10.7 (7.6, 13.8)	1.5 (0.5, 2.6)	1.4 (0.4, 2.5)	5.7 (3.0, 8.4)	3.2 (1.7, 4.8)
4-10	2.9 (1.9, 4.0)	3.5 (1.6, 5.5)	4.1 (2.2, 5.9)	3.5 (2.0, 5.0)	4.7 (3.2, 6.2)	3.7 (1.6, 5.7)	5.9 (3.3, 8.4)	6.2 (4.0, 8.4)
11-18	2.0 (1.0, 3.0)	1.5 (0.3, 2.7)	2.1 (0.8, 3.4)	4.7 (2.8, 6.5)	2.6 (1.5, 3.6)	1.5 (0.2, 2.9)	3.4 (1.5, 5.3)	6.6 (4.2, 9.0)
19-64	4.2 (3.1, 5.2)	5.0 (3.4, 6.7)	8.0 (6.0, 10.0)	7.2 (5.6, 8.8)	3.6 (2.6, 4.6)	5.4 (3.6, 7.2)	9.6 (7.3, 11.9)	10.4 (8.5, 12.3)
≥65	1.2 (0.1, 2.2)	2.4 (0.6, 4.1)	2.8 (1.0, 4.6)	3.4 (1.4, 5.4)	1.8 (0.7, 3.0)	3.4 (0.9, 6.0)	2.8 (0.3, 5.3)	6.2 (3.6, 8.9)
Sex								
Male	2.5 (1.6, 3.4)	2.6 (1.2, 3.9)	4.8 (3.1, 6.5)	4.5 (3.1, 5.8)	2.7 (1.8, 3.6)	4.1 (2.2, 6.0)	6.4 (4.3, 8.5)	8.0 (6.2, 9.9)
Female	4.2 (3.1, 5.3)	5.5 (3.8, 7.2)	7.4 (5.4, 9.5)	7.6 (5.9, 9.2)	3.8 (2.8, 4.7)	4.7 (3.2, 6.2)	8.3 (5.9, 10.6)	9.3 (7.4, 11.2)
Race								
White	3.3 (2.6, 4.0)	4.1 (2.9, 5.3)	6.0 (4.6, 7.4)	5.7 (4.6, 6.8)	1.9 (1.4, 2.4)	2.8 (1.7, 3.9)	5.6 (4.2, 7.1)	7.9 (6.5, 9.2)
Other*	3.9 (1.7, 6.1)	3.8 (1.6, 6.1)	6.9 (2.8, 11.0)	7.9 (4.4, 11.5)	13.0 (9.2, 16.8)	16.7 (11.0, 22.4)	16.5 (10.7, 22.4)	13.7 (9.4, 17.9)
Income Tertile**								
Low	1.9 (0.8, 3.0)	2.5 (1.1, 3.9)	4.3 (2.0, 6.5)	3.8 (2.0, 5.6)	2.7 (1.6, 3.9)	5.3 (2.6, 8.0)	5.0 (2.8, 7.3)	8.0 (5.6, 10.5)
Medium	2.7 (1.6, 3.9)	4.3 (1.9, 6.8)	4.1 (2.0, 6.2)	5.7 (3.7, 7.7)	2.7 (1.7, 3.7)	1.6 (0.4, 2.8)	6.6 (3.6, 9.7)	7.4 (5.1, 9.7)
High	5.9 (4.3, 7.4)	5.7 (3.6, 7.8)	9.3 (6.7, 12.0)	11.3 (8.6, 14.0)	4.3 (3.0, 5.6)	5.5 (3.3, 7.8)	8.9 (6.1, 11.7)	8.1 (5.9, 10.2)
Education***								
Low(≤16 y)	1.6 (0.9, 2.4)	2.0 (0.5, 3.5)	1.8 (0.6, 2.9)	3.3 (1.8, 4.8)	1.6 (0.8, 2.4)	2.7 (1.1, 4.3)	3.2 (1.8, 4.6)	5.0 (3.1, 6.9)
Medium (17-18y)	4.2 (2.2, 6.2)	4.6 (2.0, 7.2)	7.2 (4.0, 10.5)	4.7 (2.6, 6.9)	2.9 (1.4, 4.4)	7.0 (3.0, 11.1)	5.2 (2.7, 7.7)	8.1 (5.1, 11.1)
High(≥19 y)	6.0 (4.0, 8.1)	7.8 (4.7, 11.0)	13.5 (9.6, 17.4)	10.7 (7.9, 13.4)	6.2 (4.0, 8.4)	7.7 (4.7, 10.7)	15.6 (11.1, 20.2)	15.2 (12.0, 18.4)

*Other includes Mixed ethnic group, Black or Black British, Asian or Asian British, Any other group. These groups were combined because there was not sufficient sample size in each group. ** Equivalised household income tertiles was calculated by creating a score for each household (based upon the number, age and relationships of adults and children in the household), and then dividing the total household income by this score to get an equivalised household income.

*** Education is the age when education is completed

Table S2. UK chickpea and bean consumption overall and according to socio-demographic characteristics, 2016-2019

	Consumers, % (95% confidence interval)		
	Chickpea consumers (n=363)	Bean consumers* (n=1708)	Non-bean consumers (n=1487)
Total	12.3 (10.8, 13.8)	48.1 (45.9, 50.3)	39.6 (37.5, 41.8)
Age group, y			
1.5-3	13.4 (9.9, 16.8)	41.5 (35.2, 47.7)	45.2 (38.8, 51.6)
4-10	8.8 (6.3, 11.3)	52.8 (48.5, 57.0)	38.5 (34.4, 42.5)
11-18	10.3 (7.4, 13.2)	46.2 (41.9, 50.6)	43.4 (39.2, 47.7)
19-64	14.4 (12.1, 16.6)	49.9 (46.8, 53.1)	35.7 (32.7, 38.7)
≥65	8.1 (5.2, 10.9)	41.8 (37.1, 46.4)	50.2 (45.4, 55.0)
Sex			
Male	10.9 (8.8, 12.9)	49.7 (46.4, 53.0)	39.4 (36.2, 42.7)
Female	13.7 (11.6, 15.8)	46.5 (43.6, 49.5)	39.8 (36.9, 42.6)
Race/ethnicity			
White	11.3 (9.8, 12.9)	47.3 (45.0, 49.7)	41.4 (39.1, 43.7)
Other	18.5 (13.6, 23.4)	53.0 (46.7, 59.3)	28.5 (23.0, 34.0)
Income Tertile			
Low	9.9 (7.2, 12.5)	46.7 (42.6, 50.8)	43.4 (39.4, 47.4)
Medium	10.8 (8.2, 13.5)	47.0 (42.8, 51.2)	42.2 (38.1, 46.3)
High	16.9 (13.8, 20.0)	51.4 (47.4, 55.3)	31.7 (28.2, 35.3)
Education (adults)			
Low (≥16 y)	6.5 (4.7, 8.3)	46.4 (42.9, 49.8)	47.1 (43.7, 50.6)
Medium (17-18 y)	10.5 (7.7, 22.8)	50.8 (46.3, 55.4)	38.7 (34.2, 43.2)
High (≥19 y)	19.8 (16.7, 22.8)	48.0 (44.3, 51.7)	32.2 (29.0, 35.5)

*bean consumer consumes beans but not chickpeas

Table S3. Comparison of dietary intakes among chickpea consumers, bean consumers*, and non-bean consumers, 2016-2019 (youth and adults)

	Multivariable adjusted[1] mean (95% CI)		
	Chickpea consumers (n=363)	Bean consumers (n=1708)	Non-bean consumers (n=1487)
Kcal/d	1686 (1622, 1749)	1670 (1635, 1704)	1584 (1548, 1620)[a]
Protein, g/d	66.3 (63.1, 69.5)[a,b]	67.5 (66.0, 69.0)[a]	63.6 (61.7, 65.5)[b]
Carbohydrate, g/d	206 (197, 215)[a,b]	210 (205, 215)[a]	196 (192, 201)[b]
Free sugar, g/d	45.9 (41.4, 50.4)	48.6 (46.4, 50.8)	46.7 (44.4, 49.0)
Dietary fiber g/d	21.1 (20.0, 22.3)[a]	18.0 (17.4, 18.6)[b]	14.8 (14.4, 15.2)[c]
Total fat, g/d	66.9 (63.9, 69.9)[a]	63.2 (61.6, 64.8)	60.8 (59.1, 62.6)
Saturated fat, g/d	23.5 (22.1, 24.9)	23.6 (22.8, 24.3)	23.2 (22.4, 23.9)
MUFA, g/d	24.8 (23.6, 26.1)[a]	23.2 (22.6, 23.8)	22.3 (21.6, 23.0)
PUFA, g/d	10.6 (10.0, 11.2)[a]	9.0 (8.7, 9.2)[b]	8.2 (7.9, 8.5)[c]
Potassium, mg/d	2652 (2550, 2755)	2550 (2494, 2606)	2355 (2297, 2414)[a]
Vitamin D, µg/d	4.7 (3.0, 6.5)	4.8 (3.7, 5.8)	4.4 (3.3, 5.6)
Iron, mg/d	11.9 (9.5, 14.3)[a,b]	10.9 (10.2, 11.5)[a]	9.3 (8.8, 9.8)[b]
Magnesium, mg/d	276.5 (262.4, 290.6)[a]	242.1 (235.7, 248.4)[b]	218.5 (211.1, 226.0)[c]
Sodium, mg	1873 (1769, 1978)	1921 (1871, 1971)	1707 (1653, 1761)[a]
Calcium, mg	831 (787, 874)	804 (782, 826)	762 (738, 786)[a]
Food groups			
Fruits and Vegetables (g)	306.8 (281.8, 331.8)[a]	244.8 (233.5, 256.1)[b]	221.6 (209.9, 233.2)[c]
Pulses and nuts (g)	37.0 (30.5, 43.4)[a]	26.3 (23.1, 29.5)[b]	1.9 (0.8, 2.9)[c]
Red and processed meat, g	26.4 (21.7, 31.1)[a]	44.9 (42.1, 47.8)	41.8 (38.9, 44.7)
Fish, g	15.0 (11.0, 19.1)	16.2 (14.4, 18.1)	14.2 (12.6, 15.9)
Oily fish, g	6.4 (3.4, 9.4)	6.3 (5.1, 7.4)	5.2 (4.0, 6.4)
Modified Healthy Diet Score (range 0-14)	7.4 (7.2, 7.7)[a]	6.5 (6.4, 6.6)[b]	6.1 (6.0, 6.2)[c]

*bean consumer consumes beans but not chickpeas. Differing letters indicate that values are different at the p<0.05 level across each row. If there is no letter than the value is not significantly different from any of the other values. [1]Adjusted for age group, sex, race, family income and education;

Table S4. Comparison of dietary intakes among chickpea consumers, bean consumers*, and non-bean consumers, 2016-2019 (just adults)

	Mean (95% CI)		
	Chickpea consumers (n=213)	Bean consumers (n=893)	Non-bean consumers (n=738)
Kcal/d	1860 (1806, 1914)	1830 (1779, 1878)	1708 (1667, 1749)[a]
Protein, g/d	74.7 (71.5, 77.9)	76.5 (74.5, 78.6)	70.3 (68.3, 72.4)[a]
Carbohydrate, g/d	219 (212, 226)	220 (213, 227)	202 (197, 208)[a]
Free sugar, g/d	46.0 (42.4, 49.5)	49.0 (46.2, 51.7)	47.4 (44.2, 50.5)
Dietary fiber g/d	24.4 (23.2, 25.6)[a]	20.5 (19.6, 21.3)[b]	16.6 (16.1, 17.1)[c]
Total fat, g/d	74.0 (71.3, 76.8)[a]	69.1 (67.0, 71.3)[b]	65.8 (63.8, 67.8)[c]
Saturated fat, g/d	25.4 (24.2, 26.6)	25.4 (24.4, 26.3)	24.8 (24.0, 25.7)
MUFA, g/d	27.6 (26.4, 28.9)[a]	25.4 (24.5, 26.3)[b]	24.1 (23.3, 24.9)[c]
PUFA, g/d	12.1 (11.5, 12.7)[a]	10.0 (9.6, 10.4)[b]	9.0 (8.6, 9.3)[c]
Potassium, mg/d	3023 (2928, 3118)	2906 (2832, 2980)	2656 (2590, 2722)[a]
Vitamin D, μg/d	6.8 (5.1, 8.5)	6.0 (4.5, 7.4)	5.6 (4.3, 6.9)
Iron, mg/d	14.1 (11.5, 16.7)	12.5 (11.7, 13.3)	10.9 (10.0, 11.8)[a]
Magnesium, mg/d	324.2 (309.2, 339.1)[a]	283.0 (274.1, 291.7)[b]	250.9 (242.5, 259.4)[c]
Sodium, mg	2033 (1952, 2114)	2100 (2039, 2160)	1858 (1797, 1919)[a]
Calcium, mg	862.2 (824.1, 900.3)	855.5 (826.1, 884.8)	805 (777, 832)[a]
Fruits and Vegetables (g)	387.7 (366.0, 409.3)[a]	294.7 (281.0, 308.4)[b]	255.9 (242.2, 269.7)[c]
Pulses and nuts (g)	45.5 (39.3, 51.8)[a]	31.1 (26.6, 35.7)[b]	4.1 (3.3, 4.8)[c]
Red and processed meat, g	24.3 (19.7, 28.8)[a]	48.1 (44.6, 51.5)	44.4 (41.0, 47.7)
Fish, g	20.8 (17.1, 24.6)	21.6 (19.3, 23.9)	21.8 (19.4, 24.2)
Oily fish, g	10.5 (7.6, 13.5)	9.6 (8.2, 11.1)	7.9 (6.5, 9.3)
Modified Healthy Diet Score (range 0-14)	8.0 (7.8, 8.2)[a]	6.8 (6.6, 6.9)[b]	6.2 (6.1, 6.4)[c]

*bean consumer consumes beans but not chickpeas

Table S5. Comparison of dietary intakes among chickpea consumers, bean consumers*, and non-bean consumers among adults only, 2016-2019

	Multivariable Adjusted[1] Mean (95% CI)		
	Chickpea consumers (n=213)	Bean consumers (n=893)	Non-bean consumers (n=738)
Kcal/d	1812 (1738, 1885)	1793 (1750, 1837)	1697 (1651, 1744)[a]
Protein, g/d	73.4 (69.7, 77.1)[ab]	74.8 (73.0, 76.7)[a]	70.8 (68.3, 73.4)[b]
Carbohydrate, g/d	212 (202, 223)[ab]	215 (209, 222)[a]	200 (194, 207)[b]
Free Sugar, g/d	46.4 (41.0, 51.8)	48.5 (45.7, 51.3)	46.3 (43.3, 49.4)
Dietary fiber g/d	23.6 (22.2, 24.9)[a]	20.2 (19.4, 21.0)[b]	16.7 (16.1, 17.2)[c]
Total fat, g/d	71.9 (68.3, 75.5)[a]	67.8 (65.8, 69.8)[ab]	65.1 (62.7, 67.5)[b]
Saturated fat, g/d	24.9 (23.3, 26.5)	25.0 (24.0, 25.9)	24.4 (23.4, 25.4)
MUFA, g/d	26.7 (25.1, 28.2)[a]	24.8 (24.1, 25.6)	23.9 (22.9, 24.8)
PUFA, g/d	11.6 (10.8, 12.3)[a]	9.7 (9.4, 10.1)[b]	8.9 (8.5, 9.3)[c]
Potassium, mg/d	2995 (2876, 3115)	2880 (2806, 2953)	2675 (2598, 2753)[a]
Vitamin D, μg/d	6.1 (4.0, 8.3)	6.2 (4.7, 7.6)	5.9 (4.3, 7.6)
Iron, mg/d	13.4 (10.5, 16.4)[ab]	12.3 (11.4, 13.3)[a]	10.7 (9.9, 11.4)[b]
Magnesium, mg/d	315.5 (297.7, 333.3)[a]	278.6 (270.0, 287.2)[b]	253.2 (243.1, 263.3)[c]
Sodium, mg	2033 (1914, 2153)	2070 (2007, 2133)	1840 (1770, 1911)[a]
Calcium, mg	870 (818, 922)	851 (821, 880)	799 (767, 831)[a]
Fruits and Vegetables (g)	358.9 (328.9, 388.9)[a]	294.0 (278.8, 309.2)[b]	267.8 (252.2, 283.4)[c]
Pulses and nuts (g)	40.7 (33.1, 48.3)[a]	30.0 (25.6, 34.3)[b]	4.1 (3.0, 5.2)[c]
Red and processed meat, g	28.8 (23.2, 34.3)[a]	48.6 (44.9, 52.4)	44.2 (40.5, 48.0)
Fish, g	19.9 (15.0, 24.8)	21.6 (19.0, 24.1)	19.4 (17.1, 21.7)
Oily fish, g	10.2 (6.6, 13.9)	10.1 (8.5, 11.7)	8.7 (7.0, 10.4)
Modified Healthy Diet Score (range 0-14)	7.7 (7.4, 8.0)[a]	6.8 (6.6, 6.9)[b]	6.2 (6.1, 6.4)[c]

*bean consumer consumes beans but not chickpeas. Differing letters indicate that values are different at the p<0.05 level across each row. If there is no letter than the value is not significantly different from any of the other values. [1]Adjusted for age group, sex, race, family income and education;
